# Defect-mediated phonon dynamics in TaS_2_ and WSe_2_

**DOI:** 10.1063/1.4982817

**Published:** 2017-05-01

**Authors:** Daniel R. Cremons, Dayne A. Plemmons, David J. Flannigan

**Affiliations:** Department of Chemical Engineering and Materials Science, University of Minnesota, 421 Washington Ave. SE, Minneapolis, Minnesota 55455, USA

## Abstract

We report correlative crystallographic and morphological studies of defect-dependent phonon dynamics in single flakes of 1T-TaS_2_ and 2H-WSe_2_ using selected-area diffraction and bright-field imaging in an ultrafast electron microscope. In both materials, we observe in-plane speed-of-sound acoustic-phonon wave trains, the dynamics of which (i.e., emergence, propagation, and interference) are strongly dependent upon discrete interfacial features (e.g., vacuum/crystal and crystal/crystal interfaces). In TaS_2_, we observe cross-propagating in-plane acoustic-phonon wave trains of differing frequencies that undergo coherent interference approximately 200 ps after initial emergence from distinct interfacial regions. With ultrafast bright-field imaging, the properties of the interfering wave trains are observed to correspond to the beat frequency of the individual oscillations, while intensity oscillations of Bragg spots generated from selected areas within the region of interest match well with the real-space dynamics. In WSe_2_, distinct acoustic-phonon dynamics are observed emanating and propagating away from structurally dissimilar morphological discontinuities (vacuum/crystal interface and crystal terrace), and results of ultrafast selected-area diffraction reveal thickness-dependent phonon frequencies. The overall observed dynamics are well-described using finite element analysis and time-dependent linear-elastic continuum mechanics.

## INTRODUCTION

The pioneering work by Zewail and co-workers on the development of femtosecond (fs) transmission electron microscopy (TEM) (i.e., ultrafast electron microscopy, UEM) has led to many contributions to the understanding of ultrafast structural and electronic dynamics in materials.^1–3^ More specifically, because UEM is essentially an expansion of conventional TEM into the ultrafast temporal regime (with current instruments comprised of commercially available technologies), fs structural studies can be conducted in both real and reciprocal space with this approach.^4–7^ As is common with dedicated ultrafast electron diffraction instruments,^8–17^ UEM parallel-beam diffraction has been used to probe reciprocal-space dynamics of structurally ordered materials, with the results being interpreted within the context of the (spatially averaged) photoinduced response of interplanar spacings, variation in atomic-vibration amplitudes and reciprocal-lattice orientations, and changes in unit-cell symmetries.^18–20^ Owing to the illumination system and scan coils, however, UEM has the added capability of the formation of nanoscale probes ideally suited for elucidating dynamics within specific specimen regions of interest via convergent-beam and scanning UEM.^21–24^

An especially useful capability of UEM is the ability to select either the back focal plane or the image plane of the objective lens as the object of the first intermediate lens in the imaging (projection) system, as is routine with conventional TEM. In this way, one can correlate both reciprocal- and real-space information from specimen regions of interest, in a straightforward manner, in order to develop a comprehensive picture of ultrafast atomic to mesoscale structural and morphological dynamics.^25^ Indeed, this approach has been used to study a variety of materials phenomena, including transient elastic lattice deformation, photomechanical motion, and single-particle photoswitching dynamics.^26–30^ Owing to the aperiodic arrangement and (often) low number density of defects and interfaces relative to angstrom-scale spatial periodicities, the dynamics of energy nucleation, transport, and conversion in the presence of such features are especially well-suited to study with the combined nanoscale real- and reciprocal-space capabilities of UEM.^31,32^

Here, the detailed, spatially varying information that can be extracted with correlated UEM imaging and diffraction is demonstrated via isolation and quantification of morphologically dependent acoustic-wave dynamics in many-layer specimens of the transition metal dichalcogenides (TMDs) 1T-TaS_2_ and 2H-WSe_2_. Both materials are crystallographically similar to graphite in their layered structure, though here the layers are molecular (units of TaS_2_ and WSe_2_), not atomic. In addition, 1T-TaS_2_ exhibits trigonal symmetry, while 2H-WSe_2_ has hexagonal symmetry. Interest in these materials, as well as many other TMDs in general, stems largely from observations of tunable optical, thermal, and mechanical properties with layer number.^33^ For TMDs such as TaS_2_, which display charge-density waves and first-order transitions in electrical conductivity, open questions relating to modulation of electron density and accompanying lattice distortion could potentially be clarified with correlated ultrafast structural and electronic experimentation.^12,34–36^ For semiconductor TMDs such as WSe_2_, modulation of band-gap energy and *k*-vectors with layer number makes such materials ideal for studying coherent excitonic phenomena (e.g., coupling of excitons to optical- and acoustic-phonon reservoirs) that serve as the basis for optoelectronic applications.^37–41^

For the work reported here, coherent acoustic-phonon dynamics were induced in freestanding, many-layer flakes via fs optical excitation. For both TaS_2_ and WSe_2_, it is observed that boundary conditions imposed by specimen morphology lead to multiple in-plane acoustic modes, which exhibit robust and quantifiable interference effects (e.g., beating), the time-dependent behavior of which is largely dependent upon the relative spatial arrangement of extended defects and interfaces. As previously demonstrated, the in-plane waves are observable with UEM real-space imaging via transient and propagating diffraction-contrast features arising from spatial variation in the Bragg condition caused by local elastic strain of the lattice.^31^ Here, changes in the Bragg condition were confirmed and monitored with fs selected-area diffraction experiments on the corresponding regions of interest in which real-space dynamics were observed. Accordingly, correlating real- and reciprocal-space dynamics enabled formulation of an atomic to mesoscale picture of the evolution of acoustic-wave dynamics. For WSe_2_, for example, quantification of angular and scattering-vector-magnitude diffraction-spot dynamics suggests an initial interlayer expansion that produces coherent, compressional waves that propagate along the *c*-axis and oscillate between the outer layers of the crystal. This motion then rapidly couples to in-plane modes from the resultant differential stresses arising at distinct interfaces (e.g., vacuum/crystal and crystal/crystal). A finite-element simulation using a time-dependent linear-elastic model supports this hypothesis and yields further insight into the nature of acoustic-phonon dynamics in many-layer materials.^42^

## METHODS

### Specimen preparation

Many-layer specimens were prepared from bulk crystals of 1T-TaS_2_ (HQ Graphene) and 2H-WSe_2_ (Nanoscience Instruments, Inc.) following a previously described method.^31^ The first step in this method is isolation of many-layer flakes from bulk crystals via mechanical exfoliation with adhesive tape. In order to further reduce the number of layers, additional exfoliation steps are applied to the many-layer flakes until the approximate desired thickness is achieved, as determined by the onset of optical transparency. The flakes are then transferred onto cleaved NaCl crystals (Ted Pella, Inc.) and washed three times with isopropyl alcohol (IPA). A 4-wt% solution of poly(methyl methacrylate) (PMMA) in anisole is then drop-cast onto the flakes and allowed to cure at 95 °C for 10 min. The NaCl/TMD/PMMA stack is then placed in a deionized-water bath until the NaCl is completely dissolved. The specimens are then transferred to an IPA bath in a watch glass and floated onto a 2000-mesh Cu TEM grid (Ted Pella, Inc.). The PMMA backing layer is then removed via several washes with warm acetone, and the specimens are dried in air at 85 °C for 2 h. The as-prepared specimens generally consist of heterogeneously distributed freestanding flakes of varying thicknesses.

### Laser system

Specimens were excited *in situ* with 700-fs pulses (full-width at half-maximum, FWHM, as measured with an in-house-built autocorrelator) from a diode-pumped, ytterbium-doped potassium gadolinium tungstate (1030 nm fundamental) solid-state laser (PHAROS, Light Conversion). The repetition rate of the laser was selected according to the UEM experimental requirements for each specimen; generally, a compromise must be struck between photoelectron beam current, pulse-to-pulse specimen recovery, and sufficient excitation within the region of interest.^43^ Here, repetition rates of 67 and 25 kHz were used for the TaS_2_ and WSe_2_ experiments, respectively. All experiments were performed at ambient conditions. Owing to the repetition rates used, a modestly elevated pulse-to-pulse steady-state temperature is expected, which in the case of TaS_2_ is expected to be above the nearly commensurate to incommensurate charge-density-wave transition temperature (350 K).^44^ The laser wavelengths used to excite the specimens were 1030 and 515 nm (TaS_2_ and WSe_2_, respectively). The excitation spot size at the specimen position was 100 *μ*m FWHM, as estimated from *ex situ* measurements, resulting in pump fluences of 3.0 and 0.5 mJ/cm^2^ for TaS_2_ and WSe_2_, respectively. As is generally done, fourth-harmonic ultraviolet (257.5 nm) laser pulses were used to generate the discrete photoelectron packets, with laser-pulse durations assumed to be approximately equivalent to the fundamental and second-harmonic pump pulses.^43^

### Ultrafast electron microscope

The UEM at Minnesota is an FEI Tecnai Femto (Thermo Fisher Scientific), typically operated at 200 kV in both thermionic (for identification and initial characterization of suitable specimen regions) and pulsed-photoelectron modes. Owing to additional unrelated work in the lab, several different types of LaB_6_ cathodes were ultimately used to generate the data reported here: experiments on TaS_2_ were performed using a 50-*μ*m flat, graphite-encircled LaB_6_ cathode (Applied Physics Technologies), while the WSe_2_ experiments used both the aforementioned cathode and a 150-*μ*m flat, graphite-encircled cathode. Preliminary results indicate that the presence of the graphite improves the overall beam-current stability during UEM operation and also eliminates photoemission from the LaB_6_ shank. Custom Wehnelt (1.5-mm diameter) and condenser (1.25-mm diameter) apertures were used to increase the total beam current reaching the specimen in the photoelectron mode. Images were recorded with a Gatan Orius SC200B 4 megapixel fiber-coupled CCD camera (14-bit dynamic range), with individual-image acquisition times ranging from 25 to 30 s. Electrons per photoelectron packet were estimated to range from 250 to 1000, resulting in a temporal instrument response ranging between 3 and 4 ps FWHM, respectively, for the experimental settings used here, as estimated from previous measurements.^43^

## RESULTS AND DISCUSSION

### Cross-propagating acoustic waves in TaS_2_

**FIG. 1.**
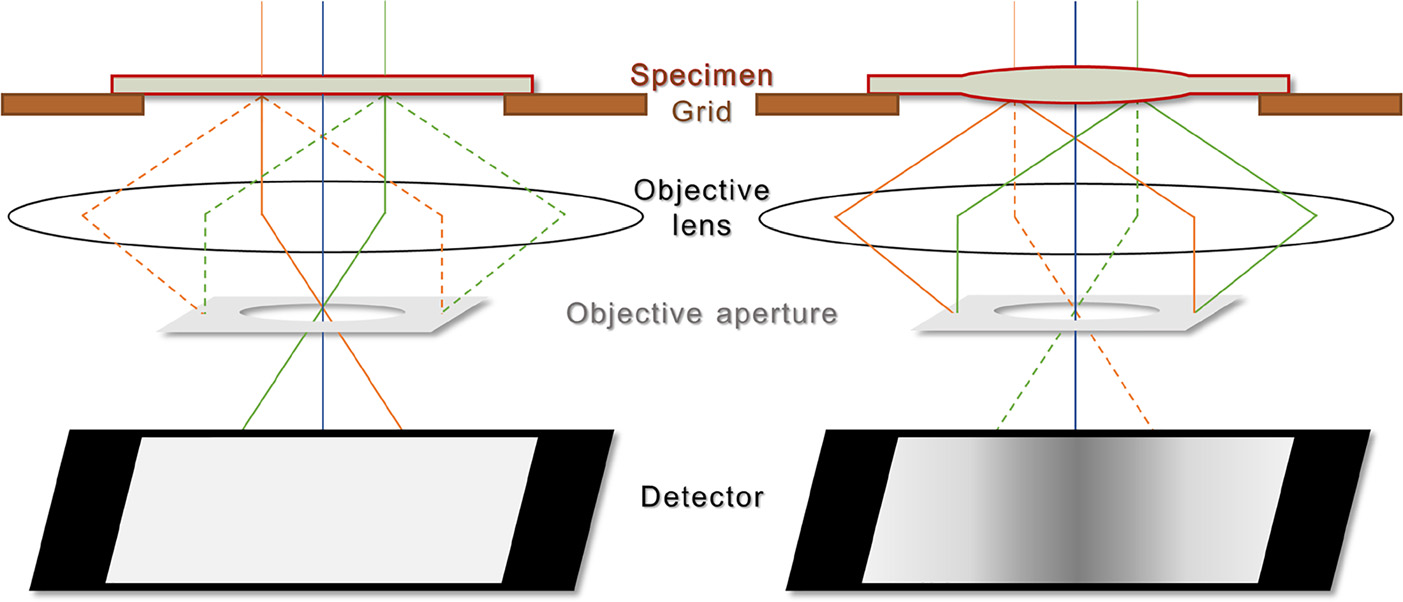
Simplified schematic of the UEM imaging-contrast mechanism arising from transient local elastic strain. (Left) Freestanding specimen prior to laser-induced deformation. Under this condition, the diffracted beams (green and orange dashed rays) are only weakly excited, and, thus, the resulting image contrast is weak owing to most incident electrons being contained in the direct beam (green and orange solid rays; blue solid line is the optic axis); the direct beam is selected for image formation with the objective aperture. (Right) Upon photoexcitation, elastic deformation of a properly oriented specimen produces a spatially varying orientation, with some regions satisfying the Bragg condition, thus leading to strong excitation of the diffracted beams (green and orange solid rays). This produces a relative reduction in the number of incident electrons contained in the direct beam (green and orange dashed rays) and an increase in the image-contrast strength. As the diffraction-contrast strength is a function of the strain, the strongest image contrast arises from the most highly strained specimen region; in this schematic, that specimen region is on the optic axis (i.e., the blue solid line). The black regions at the edges of the detector represent the electron-opaque TEM grid bars.

Imaging of acoustic-wave dynamics using stroboscopic UEM is enabled by the sensitivity of electron-scattering vectors to slight, time-varying orientations of the crystal lattice, in much the same way as occurs in static, conventional TEM diffraction-contrast imaging of bend contours. As photogenerated lattice waves travel across the imaging field of view, the atomic planes respond via local deformation, which causes tilting and oscillation of the (reciprocal) lattice with respect to the fixed Ewald sphere. This produces a corresponding local modulation of the Bragg condition. By inserting an aperture into the back focal plane of the objective lens, strongly scattered electrons emerging from specimen regions satisfying the Bragg condition are blocked from entering the imaging (projection) system (Fig. 1). Accordingly, the specimen region from which this scattering occurs appears dark in the resultant bright-field image, and the propagating lattice waves manifest as coherent, propagating contrast waves in UEM videos.^31^ Further, it is expected that the degree of lattice deformation will manifest as variations in contrast strength; that is, larger local elastic strains should produce stronger contrast, depending upon the initial crystal orientation with respect to strongly scattering conditions.

**FIG. 2.**
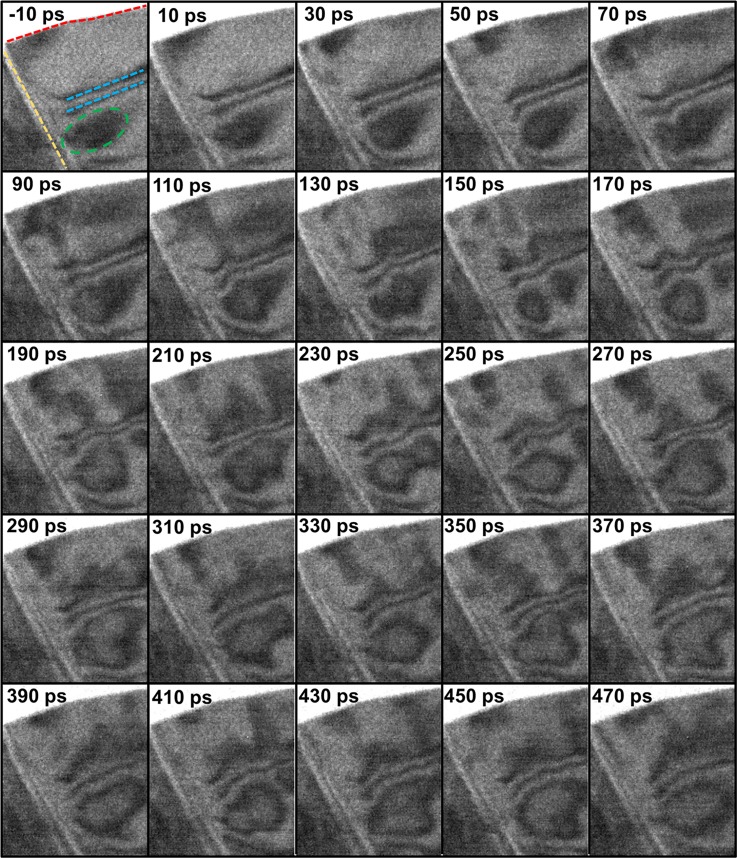
Select frames from a stroboscopic UEM image series showing photoexcited, cross-propagating acoustic-wave trains in a freestanding, many-layer TaS_2_ flake. Each frame is 413 by 486 pixels, with a field-of-view of 1.32 by 1.56 *μ*m. The red and yellow dashed lines denote the vacuum/crystal and crystal/crystal interfaces, respectively. The green dashed ellipse and blue dashed lines denote diffraction-contrast features that were modulated by the propagating acoustic waves and monitored to quantify dynamics. For the image series, 30-s acquisition times per frame were used, and the entire scan ranged from −150 to 475 ps. The temporal step-size was 4 ps from −150 to −25 ps and 2 ps from −25 to 475 ps. The step-size was varied in order to reduce the total experiment time while still capturing the full dynamic information of interest following photoexcitation. The figure is comprised of select frames at 20-ps intervals, ranging from −10 to 470 ps (time stamps are in the upper-left corner of each frame). The specimen was oriented approximately along the [001] zone axis, and all images were obtained with a 20-*μ*m objective aperture in order to select the direct beam for imaging. The corresponding UEM video, from which the static image was generated, was acquired with a repetition rate of 67 kHz. (Multimedia view) [URL: http://dx.doi.org/10.1063/1.4982817.1]
10.1063/1.4982817.1

With UEM bright-field imaging at sufficient magnifications, the roles of individual defects on lattice-wave nucleation and propagation can be directly determined. Shown in Figure 2 is the photoexcited emergence, propagation, and interference of two perpendicularly oriented coherent acoustic-wave trains observed emanating from two distinct interfaces (vacuum/crystal and crystal/crystal) in many-layer TaS_2_. The UEM image series, as acquired, contains a total of 136 images, a select few of which comprise Figure 2 (Multimedia view). The first observed acoustic wavefronts emerge at early times (i.e., before 70 ps), seemingly simultaneously from the vacuum/crystal and crystal/crystal interfaces, and propagate along wave vectors oriented normal to each interface. Additional wavefronts continue to emerge from these regions and follow the same initial wave vectors for hundreds of picoseconds. Here, the wave trains can be visualized by following the temporal response of initially static diffraction-contrast features (i.e., those marked with the blue dashed lines and green dashed ellipse in the −10-ps frame of Fig. 2). Owing to the relative orientations of the wave vectors, coherent interference effects are observable at time delays beyond 200 ps. Prior to this, interference effects are modulated primarily by the wave train emanating from the crystal/crystal interface.

**FIG. 3.**
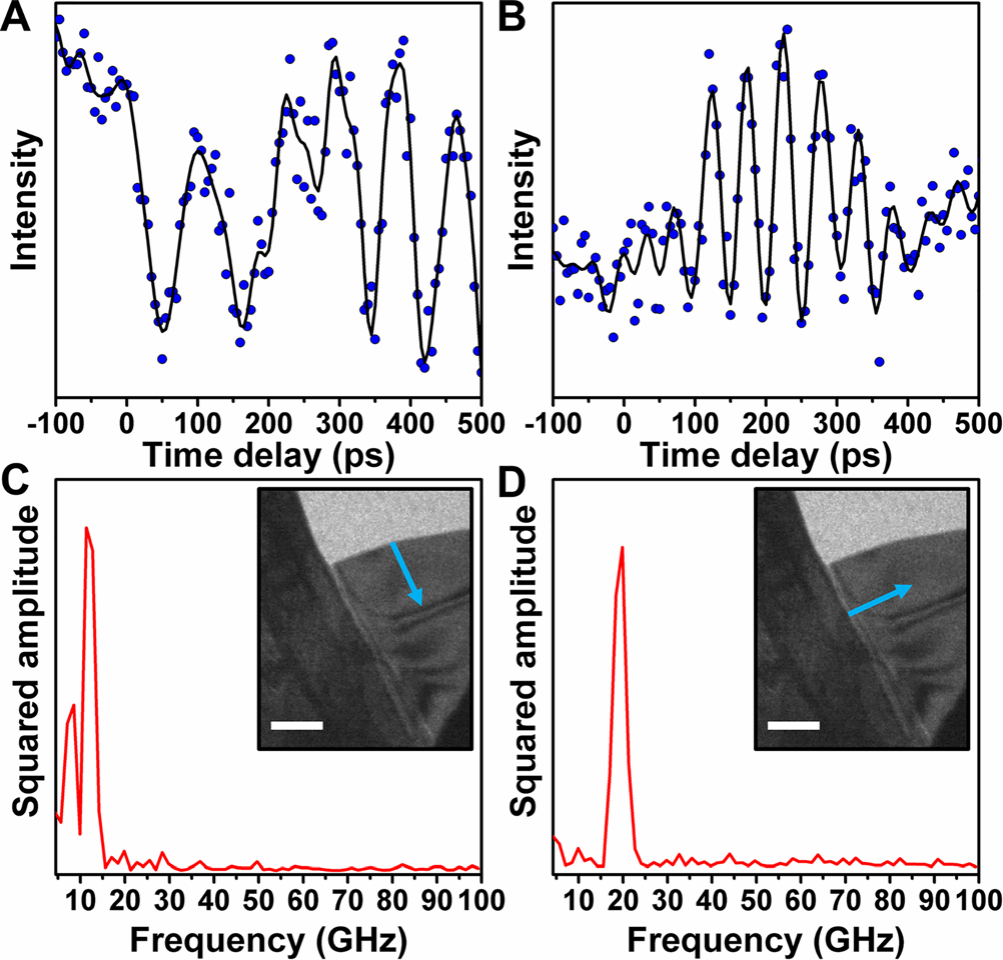
Cross-propagating acoustic phonons in a TaS_2_ flake. (a) Time-dependent image intensity obtained from a 20 by 20-pixel region of interest in which the primary dynamic contrast behavior arises from acoustic-wave propagation normal to the vacuum/crystal interface, as denoted by the light-blue arrow in the inset of panel (c). (b) Time-dependent image intensity as in panel (a), but obtained from a region of interest in which the primary dynamic contrast behavior arises from acoustic-wave propagation normal to the crystal/crystal interface, as denoted by the light-blue arrow in the inset of panel (d). The solid black curves in (a) and (b) are smoothed data, generated with a low-pass filter (30-GHz cutoff), to guide the eye. (c) Time-domain Fourier transform of the temporal trace in panel (a), with the primary contrast-wave frequencies centered between 8 and 12 GHz. (inset) Representative bright-field UEM image. The light-blue arrow denotes the direction of propagation of acoustic waves having frequencies between 8 and 12 GHz. (d) Time-domain Fourier transform of the temporal trace in panel (b), with the primary contrast-wave frequency occurring at 20 GHz. (inset) Representative bright-field UEM image. The light-blue arrow denotes the direction of propagation of acoustic waves having frequencies of 20 GHz. The scale bars represent 500 nm in both insets.

In order to quantify the wave-interference effects driven by interfaces in the TaS_2_ flake, modulated image intensity was spatially monitored as a function of time, as summarized in Figure 3. Because the onset of wave-train interference renders continued isolation and monitoring of discrete phonon properties in those particular specimen regions challenging, dynamics were quantified in areas where a single, unperturbed wave train was still observable. Here, the onset of interference was again observed to occur at times beyond 200 ps, where a discontinuity in the image-contrast oscillations, arising from the wave train emanating from the vacuum/crystal interface, can be seen [Fig. 3(a)]. A frequency analysis of the individual wave trains [Figs. 3(c) and 3(d)] also reveals the presence of interference effects, in addition to fundamental differences likely arising from dissimilar boundary conditions (e.g., differences in the mechanical properties of the discrete interfaces from which they emerge). Because the wave train emanating from the vacuum/crystal interface is disrupted due to interference, a Fourier analysis of the data [Fig. 3(c)] broken into two time windows (pre- and post-interference, centered at 250 ps) reveals the presence of two distinct frequencies: one at 8 GHz (*f_2_*) and one at 12 GHz. Because the 8-GHz frequency arises from the pre-interference dynamics (i.e., dynamics before the onset of interference), the post-interference frequency at 12 GHz is assigned as the beat frequency (*f_b_*) of the two wave trains, such that *f_b_* = *f_1_* − *f_2_*. Analysis of the wave train emanating from the crystal/crystal interface [Fig. 3(d)] returns a single frequency (*f_1_*) centered at 20 GHz.

It is hypothesized that the mechanism by which the observed cross-propagating, transverse (in-plane) waves are generated via photoexcitation is conversion of longitudinal modes at the flake surfaces due to spatially varying elastic (acoustic) properties at the interfaces.^45,46^ At the near-room-temperature conditions of the experiments, the photogeneration of coherent acoustic phonons in TaS_2_ is ascribed to the interplay of deformation potential and thermoelasticity on ultrafast timescales.^47^ As previously noted, the waves do not originate randomly in the flake, but instead are coherently generated from two distinct and structurally dissimilar interfaces. Here, the spot size of the excitation laser (100 *μ*m FWHM) is such that the spatial excitation-intensity profile is flat across the entire UEM imaging field of view. Accordingly, instantaneous excitation (relative to the in-plane phonon periods) will produce a photothermal gradient initially varying only along the *c*-axis stacking direction owing to thickness-dependent attenuation of the excitation pulse. Phonon motion that then occurs along the *c*-axis stacking direction will couple into transverse, in-plane modes in regions where bonding discontinuities occur (e.g., vacuum/crystal and crystal/crystal interfaces) owing to spatial variations in constitutive relations of the defect-laden specimen (i.e., spatial variations in the values of the governing equations of the linear elastic response).

The disparity in observed frequencies for each of the wave trains (8 and 20 GHz) is hypothesized to arise from two potential sources. First, the disparity could arise from differences in the relative crystallographic orientation of each flake region; if the wave trains travel along different crystallographic directions, then differences in elastic response may arise owing to off-diagonal elements of the TaS_2_ anisotropic strain tensor. Here, by correlating crystallographic orientation (determined with selected-area diffraction) to the observed real-space wave vectors, it was found that the wave train emanating from the vacuum/crystal interface travels approximately along the 010 direction. As expected from the UEM imaging studies, the wave train emanating from the crystal/crystal interface propagates along a different direction and approximately follows the 210 direction. This description is in reasonable agreement with previous studies of photoexcited acoustic phonons.^48,49^ The second source may be relative differences in layer number (i.e., flake thickness) for each of the regions of interest. In this case, initially excited longitudinal modes would experience different round-trip times, which is reasonably expected to cause a frequency variation of the in-plane modes upon coupling and conversion. Note that the measured velocity of the in-plane waves emanating from both interfaces is approximately 5 nm/ps, which matches well with Lamb-type plate waves in thin Si membranes.^50^ Interestingly, the interplay of Rayleigh-type lattice waves with charge-density waves in TaS_2_ has been described, and it is expected that the correlative UEM imaging and diffraction methods are well-suited to experimentally probe this phenomenon.^51^

### Ultrafast selected-area diffraction in the wave-interference region

**FIG. 4.**
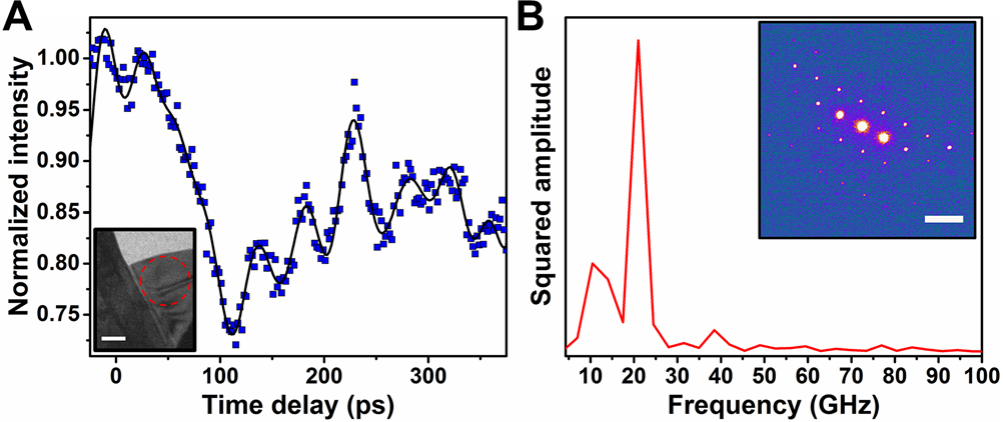
Selected-area diffraction from a region of interest in a TaS_2_ flake displaying cross-propagating acoustic-wave dynamics. (A) Time-dependent intensity of the (100) Bragg spot, as quantified by fitting with a 2D Gaussian peak function. The solid black curve are smoothed data, generated with a low-pass filter (30-GHz cutoff), to guide the eye. (inset) Representative bright-field UEM image. The red-dashed circle denotes the position and effective size of the selected-area aperture used to generate the UEM parallel-beam diffraction patterns. The scale bar represents 500 nm. (b) Time-domain Fourier transform of the temporal trace in panel (a), revealing frequencies occurring at approximately 12 and 20 GHz. (inset) Representative (false-colored) UEM selected-area diffraction pattern obtained approximately along the [001] zone axis. The scale bar represents 5 nm^−1^.

To further compare the observed real-space phonon dynamics to modulations at the crystal-lattice level, UEM selected-area diffraction was conducted in the same specimen region, with all other experimental parameters (e.g., pump laser fluence, spot size, and excitation wavelength) held constant. Figure 4 summarizes the results of the diffraction study, wherein the modulation of Bragg spot intensity was monitored as a function of time. By using a selected-area aperture to isolate a region of interest within which each wave train, as well as the subsequent interference effects, was observed in real space, the reciprocal-space response—which is a spatial average of all periodic-structural dynamics occurring within this specimen region—can be more-clearly understood. For example, by monitoring the (100) reflection, which displayed the strongest time-dependent intensity modulation (e.g., a nearly 30% decrease in intensity during the first 100 ps), both the wave train emanating from the vacuum/crystal interface and the onset of interference were detected. Specifically, an approximately 20-GHz intensity oscillation sets in following the initial intensity drop, which matches well with the single wave train (*f_1_*) emanating from the vacuum/crystal interface. This oscillation then displays a variation in both amplitude and frequency at approximately 250 ps, which matches well both spatially and temporally with the onset of wave interference, as directly observed with UEM real-space imaging described above. The Fourier transform of this entire time trace also displays a lower-amplitude oscillation at approximately 12 GHz, which is likely a convolution of both the lower-frequency wave train emanating from the crystal/crystal interface (*f_2_*) and the onset of the beat frequency (*f_b_*).

### Ultrafast bright-field imaging of WSe_2_ flakes

**FIG. 5.**
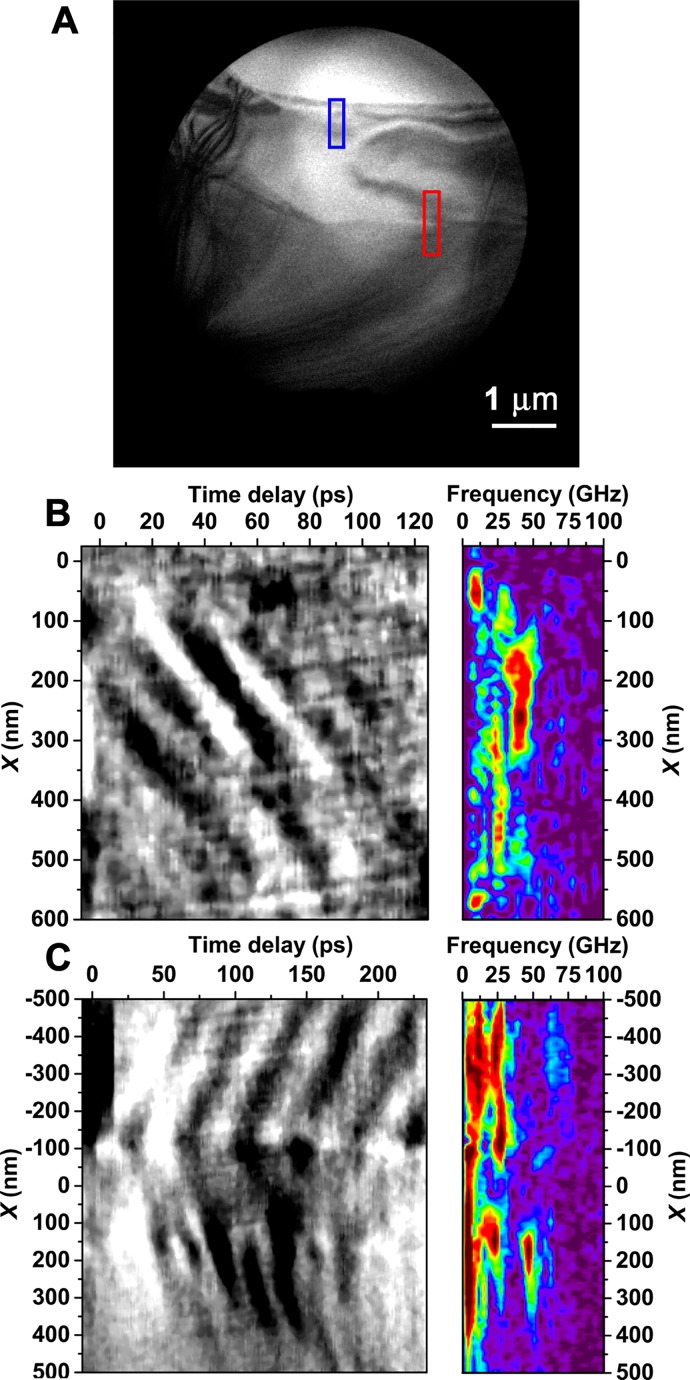
Quantification of propagating acoustic waves in a freestanding WSe_2_ flake from a UEM bright-field image series. (a) Representative bright-field UEM image of a WSe_2_ flake, with two distinct interfaces highlighted: the vacuum/crystal interface (blue rectangle) and a terraced interface (red rectangle). The black circle around the periphery of the image is due to an inserted selected-area aperture. (b, left) Space-time contour plot generated from the vacuum/crystal interfacial region highlighted with the blue rectangle in panel (a). Each time point was generated by first averaging image counts along the horizontal direction of the region of interest and then subtracting pre-time-zero values to isolate the relative change. For reference, the 0-nm spatial position corresponds to the vacuum/crystal interface. (b, right) Fourier transform of the temporal traces generated by analyzing the space-time contour plot. The frequency of the mode emanating from the vacuum/crystal interface is approximately 40 GHz. (c, left) Space-time contour plot generated from the terraced interfacial region highlighted with the red rectangle in panel (a). For reference, the 0-nm spatial position corresponds to the approximate position of the terrace in the region of interest. (c, right) Fourier transform of the temporal traces revealing the presence of an acoustic wave of approximately 25 GHz emanating and propagating away from the terrace, with an apparent double resonance of this mode (50 GHz) appearing in the thicker region of the crystal (i.e., lower region of the image). The corresponding UEM video was acquired with a 25-kHz repetition rate and a 25-s acquisition time per frame. The video illustrates dynamics at 1-ps steps spanning from −5 to 236 ps (241 total images) and plays at 60 fps. The scale bar in the video represents 1 *μ*m. (Multimedia view) [URL: http://dx.doi.org/10.1063/1.4982817.2]
10.1063/1.4982817.2

Similar propagating contrast features were observed in many-layer flakes of WSe_2_. Like TaS_2_, WSe_2_ has a layered atomic structure with strong covalent in-plane bonding and weak van der Waals bonding between layers. Upon thinning to a few layers, WSe_2_ transitions from an indirect to direct band gap, making it an appealing candidate for next-generation optoelectronic applications.^37,52,53^ Here, however, the flakes are expected to display behaviors more-closely matching those of the bulk regime, which exhibits an indirect band gap of approximately 1.3 eV.^54^ As with the TaS_2_ specimen described above, both UEM bright-field imaging and selected-area diffraction were conducted on a single flake of WSe_2_, the results of which were correlated in order to develop a comprehensive atomic-to-mesoscale picture of the photoexcited structural dynamics. An overview of the WSe_2_ specimen, and the observed real-space structural dynamics, is provided in Figure 5 (Multimedia view). Following fs excitation at 515 nm (2.4 eV), two distinct acoustic-phonon behaviors are discernible with UEM bright-field imaging. In this case, the distinct behaviors are differentiated by the dissimilar morphological features from which they emerge; one wave train is observed emanating from a vacuum/crystal interface, while the other emerges from a terraced (i.e., crystal/crystal) interfacial region. As with the TaS_2_ specimen, each wave train displays a distinct oscillation frequency; 40 and 25 GHz for the vacuum/crystal and terraced wave trains, respectively [Figs. 5(b) and 5(c), respectively]. Notably, the wave trains are most apparent in regions where contrast strength sharply varies spatially. Accordingly, small angular modulations in local lattice orientation, relative to the fixed Ewald sphere, due to relatively low-energy acoustic-phonon propagation give rise to discernible diffraction-contrast dynamics in the UEM images series.^7,19,28,31^

The behavior of the WSe_2_ contrast dynamics at each interface can be quantified by defining a region of interest, generating line scans parallel to the propagation direction (by averaging image intensity orthogonal to the propagation direction), and plotting the resulting profiles as a function of time. This approach is used to produce space-time contour plots displaying coherent contrast oscillations arising from the phonon wave trains [Figs. 5(b) and 5(c)]. From such plots, one can identify both the propagation velocity of each wavefront (slope of the contrast features) and the spatial frequency of the wave train across the region of interest. For the WSe_2_ flake studied here, the terraced interface defines a spatial thickness transition region, with the thinner region spanning from the terrace to the vacuum/crystal interface. Here, the thinner region supports the 40 GHz wave train, which propagates at approximate velocities ranging from 5.5 to 7 nm/ps (depending upon which wave front is measured). In addition, the wave train is observed to propagate away from and along a vector approximately normal to the interface and oriented toward the terrace. Conversely, the wave train emanating from the terrace displays more complex behavior; two distinct wave trains can be seen emerging from the terrace and propagating away from and along vectors approximately normal to this particular interface. This behavior is manifested as contrast features in the contour plots having comparable slopes but different signs, thus illustrating the opposite propagation directions but comparable velocities (ranging from 7 to 11 nm/ps). Beyond 100 nm from, and on either side of, the terrace, oscillation frequencies of approximately 25 GHz are observed. Interestingly, a peak at 50 GHz is clearly present in the specimen region below the terrace (relative to the image plane); it is unclear whether this is a physical splitting of the wave via propagation through a specimen feature or an artifact of the complex contrast mechanisms in that particular region.

The distinctly different wave-train frequencies in the relatively thick and thin specimen regions suggest that local properties and behaviors are dictated by distinct specimen morphologies and structural features. As put forth when discussing the TaS_2_ results above, an explanation for the origin of the in-plane modes in WSe_2_ is that initial photoexcitation and rapid *c*-axis expansion lead to excitation of transverse phonons, the nucleation and propagation direction of which is dictated by spatial discontinuities in elastic properties along particular wave vectors. Indeed, GHz oscillations in graphite, as measured with UEM, have been attributed to the propagation of longitudinal waves along the *c*-axis stacking direction and reflection at the outer layers.^19,27,28^ Similarly here, it is hypothesized that the back-and-forth motion of *c*-axis longitudinal phonons ultimately couples to in-plane propagating modes via interfacial stresses. These in-plane modes then manifest as traveling contrast features having distinct wavefronts and vectors in the UEM bright-field image series due to local modulation of the Bragg condition by the propagating elastic strain. As shown below, this hypothesis is supported by the results obtained from correlative UEM selected-area diffraction studies (as was done with TaS_2_) and linear elastic modeling.

### WSe_2_ thickness-dependent resonances in ultrafast diffraction

**FIG. 6.**
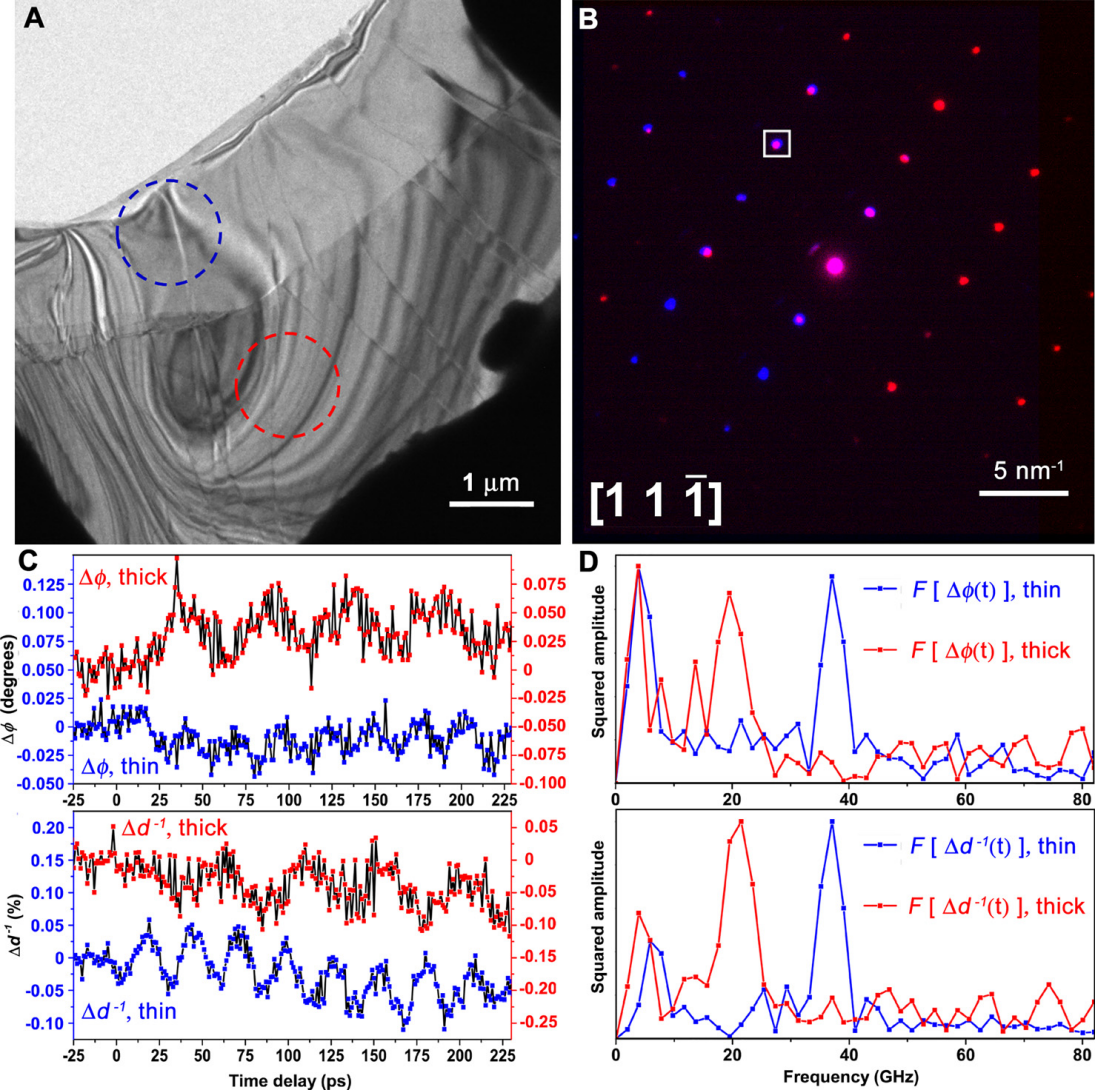
Selected-area diffraction dynamics in relatively thin and thick regions of a freestanding WSe_2_ flake. (a) Representative UEM bright-field image illustrating the relatively thin (blue dashed circle) and thick (red dashed circle) regions from which selected-area time-resolved diffraction data were obtained. (b) Composite frame consisting of UEM parallel-beam diffraction patterns acquired approximately along the $\left[11\bar{1}\right]$ zone-axis for the thin (blue) and thick (red) regions of interest; overlapping intensity between the two regions appears pink. The white square highlights the analyzed (202) reflection, which has significant diffracted-beam intensity contributed from both regions of interest. (c) Transient behavior of the azimuthal orientation (Δ*φ*, top) and diffraction-spot spacing (Δ*d*^−1^, bottom) of the (202) reflection acquired from both the thin (blue data points) and thick (red data points) specimen regions. The left (blue) and right (red) axes correspond to data from the thin and thick regions, respectively. Note that an initial contraction of the (202) diffraction-spot spacing (indicative of an initial interlayer expansion) is evident in the time trace from the thin specimen region; this initial expansion induces the oscillatory elastic response observed in both the real- and reciprocal-space UEM studies. (d) Fourier transforms of the oscillatory behavior shown in panel (c) from both the thin and thick specimen regions. Frequencies of 38 GHz in the thin region and 21 GHz in the thick region, respectively, are evident in the spectra for both Δ*φ* (top) and Δ*d*^−1^ (bottom). The observed behavior is ascribed to beating of *c*-axis longitudinal waves between the flake surfaces within regions of differing thickness.

Summarized in Figure 6 are UEM selected-area diffraction studies conducted on relatively thin and thick WSe_2_ specimen regions of interest [dashed blue and dashed red circles (corresponding to the aperture size and location), respectively, in Fig. 6(a)]. The composite representative diffraction pattern in Figure 6(b), where scattering from the thin and thick regions is mapped in blue and red RGB channels, respectively, confirms that specimen crystallinity is both continuous and oriented across the terrace interface (i.e., a single crystal), though the relative intensities of the diffracted beams vary between the regions due to crystal bending. That is, the two regions are portions of an oriented crystal rather than individual and disparate crystal grains in a polycrystalline film.

Here, with UEM, monitoring Bragg-spot properties as a function of time reveals ultrafast variations in both the spacing and azimuthal orientation of diffracted beams (relative to the direct beam). For example, Figure 6(c) summarizes the extracted dynamics of the (202) reflection, which is present in diffraction patterns from both specimen regions [white square in Fig. 6(b)]. Importantly, the onset of dynamics is observable as an initial 0.05% contraction in inverse *d*-spacing (Δ*d*^−1^) in the time scan from the thin region. The reciprocal-space contraction (i.e., real-space expansion) occurs over approximately 3 ps and is the precursor to coherent oscillations. Note that the initial temporal change is likely instrument limited at the UEM settings used here, as mentioned above. The onset of resolvable azimuthal angle resonances occurs approximately 15 ps after this initial real-space expansion, which may correspond to the first reflection of a *c-*axis longitudinal phonon from an outer crystal layer, after which oscillations in both angle and position begin and continue over the entire temporal window (here, beyond 230 ps). The observed transient modulations in diffracted-beam position indicate that the corresponding lattice planes exhibit both shear motion and lateral expansion and contraction, an effect which may stem from *a*- and *b*-axis displacements of in-plane phonons. Figure 6(d) displays Fourier transforms of both the azimuthal and radial diffraction-spot dynamics from each selected area; frequencies of 21 and 38 GHz for the thick and thin regions, respectively, are apparent in both degrees of freedom. Using the relation $T=0.5\tau \nu_{s}$, where *T* is the flake thickness, *τ* is the corresponding oscillation period (i.e., 1/*f*, where *f* is the oscillation frequency), and *ν_s_* is the speed of sound along the propagation direction (1.6 to 2.3 nm/ps for the WSe_2_ *c*-axis),^55,56^ a thickness between 20 and 30 nm is estimated for the thin region, transitioning to approximately 1.8 times that thickness across the terraced interface.

The atomic-level picture of structural dynamics revealed with ultrafast diffraction supports the hypothesis pertaining to the origins of GHz propagating, in-plane acoustic-phonon modes observed with UEM bright-field imaging. In short, an initial interlayer expansion induces an elastic response that launches compression waves that propagate along the *c*-axis layer-stacking direction. Confinement and interaction of these waves with the specimen surfaces (i.e., the outer-most specimen layers) couple to in-plane modes through differential stresses at discrete interfaces (e.g., vacuum/crystal and crystal/crystal). The physical picture of this process is explained in more detail in the next section. Note, however, that the exact and detailed nature of initial fs optical coupling to the observed thermoelastic behavior (especially with respect to distinct nanoscale defects and interfaces) is not directly addressed with the experiments reported here. Accordingly, future experiments will focus on examining timescales and amplitudes of the initial onset of dynamics as a function of photoexcited charge-carrier density and excitation wavelength in an effort to elucidate the nature of electron-phonon coupling leading to local and (eventually) global elastic deformation.

### Simulation of WSe_2_ acoustic-phonon dynamics with a linear elastic model

In order to gain further insight into the nature of the observed coherent contrast waves, a finite element simulation was performed using a transient linear elastic model with the COMSOL Multiphysics Structural Mechanics software module. Here, free boundary conditions were assumed, and the initial condition for phonon launch was an instantaneous, depth-dependent displacement along the *z*-axis (i.e., layer-stacking axis) in the crystal. This initial *z*-displacement represents the ultrafast, optically induced interlayer expansion observed in the diffraction experiments. That is, the initial condition was set to be $\Delta z\left(z,\;t=0\right)=Ae^{-\frac{z}{\alpha }}$, where *z* is the coordinate along the WSe_2_ *c*-axis, *A* is the magnitude of the initial displacement, and *α* is the inverse of the attenuation coefficient (with units of length). For illustrative purposes (exaggerated) values of *A* and *α* have been chosen to be 7.5 × 10^−2^ nm and 3.2 nm, respectively. This model was applied to an idealized mesh structure of the two regions of interest—the vacuum/crystal and terraced (crystal/crystal) interfaces—embodying a two-dimensional representation of the experimental geometry of the WSe_2_ flake. For the hexagonal symmetry of the WSe_2_ flake, five independent elastic constants make up the anisotropic elastic stiffness tensor, represented by the elasticity matrix^57^

$$C_{ij}=\left[\begin{array}{cc} \begin{array}{ccc} C_{11} & C_{12} & C_{13}\\ C_{12} & C_{11} & C_{13}\\ C_{13} & C_{13} & C_{33} \end{array} & 0\\ 0 & \begin{array}{ccc} C_{44} & 0 & 0\\ 0 & C_{44} & 0\\ 0 & 0 & \frac{C_{11}-C_{12}}{2} \end{array} \end{array}\right].$$

Here, the *C*_11_ and *C*_33_ values are 300 and 34 GPa, respectively, to match compressional speeds-of-sound along the corresponding axes [i.e., *C*_33_ = *ρ*$v_{s,c}^{2}$ = 9320 kg/m^3^·(1.9 nm/ps)^2 ^= 34 GPa], the *C*_44_ value is 19 GPa,^56^ and the *C*_12_ and *C*_13_ values are each set to 15 GPa.

**FIG. 7.**
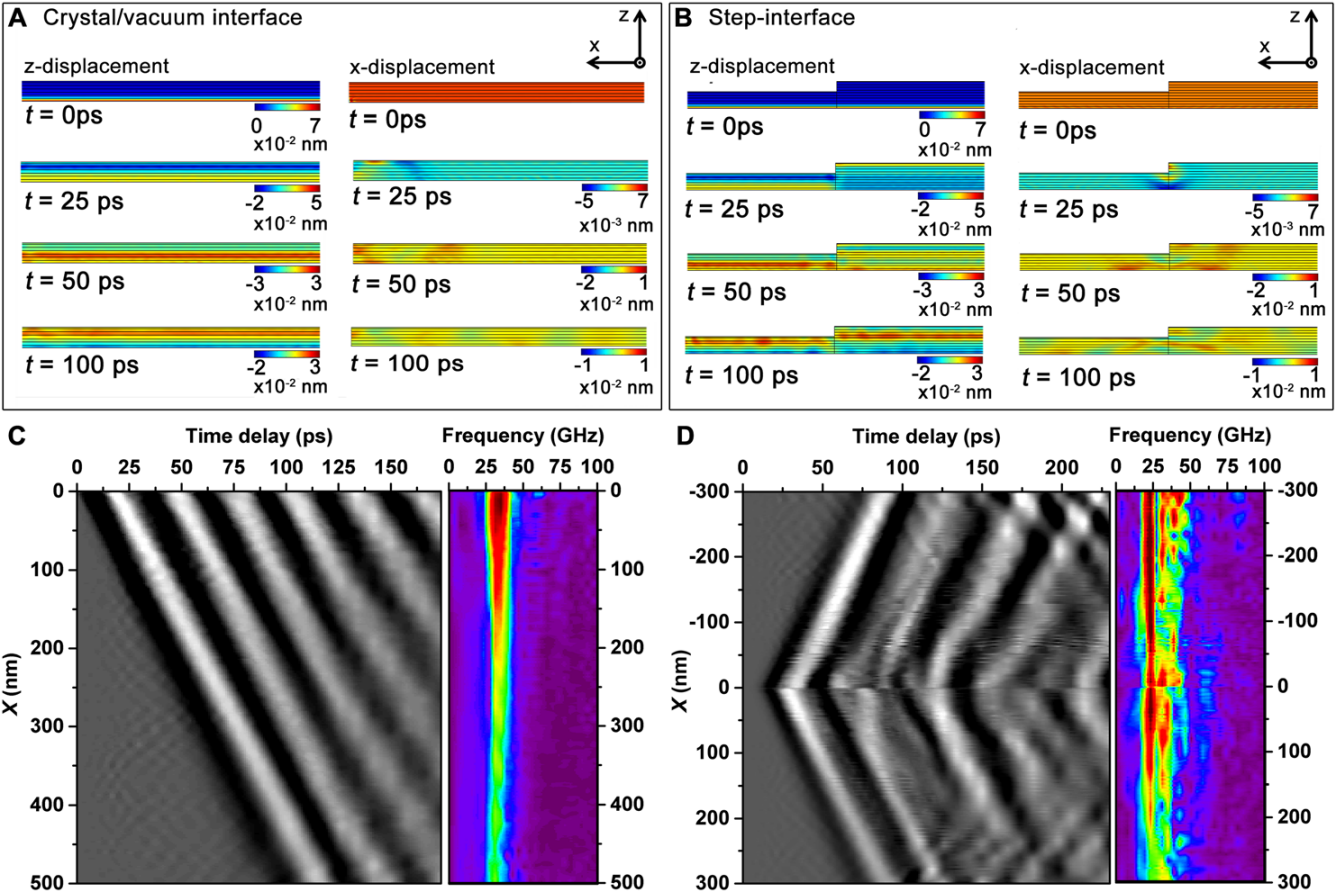
Time-dependent linear-elastic simulation of GHz propagating acoustic waves at interfaces in WSe_2_. (a) Select transients from the simulation mapping both the *z*- and *x*-displacements (i.e., the *c*-axis and in-plane directions, respectively) in regions near the vacuum/crystal interface. The corresponding video illustrates dynamics at 1-ps steps spanning from 0 to 250 ps (250 total images) and plays at 30 fps. (b) Select transients from the simulation mapping both the *z*- and *x*-displacements in regions near the terraced interface. The corresponding video illustrates dynamics at 1-ps steps spanning from 0 to 250 ps (250 total images) and plays at 30 fps. In both (a) and (b), the initial depth-dependent photoinduced expansion launches a compressional wave that oscillates along the *z*-axis. This motion couples to in-plane (i.e., *x*-axis) modes at the edge of the specimen (a) and at the terrace (b) owing to the discontinuous elastic properties at each interface. ((c) and (d), left) Space-time contour plots of the *x*-displacement generated by averaging in-plane line-scans at 3-nm intervals within the specimen. ((c) and (d), right) Space-frequency contour plots of the *x*-displacement illustrating near-quantitative agreement with experiment; behaviors that are quantitatively similar to experimental observations are captured in the simulations, including the presence of specimen-thickness-dependent frequencies and speed-of-sound wave trains emanating from the interfacial regions. (Multimedia view) [URL: http://dx.doi.org/10.1063/1.4982817.3
10.1063/1.4982817.3] [URL: http://dx.doi.org/10.1063/1.4982817.4]
10.1063/1.4982817.4

The results of the simulation are visualized in Figures 7(a) and 7(b) (Multimedia view). Each frame (A and B) represents the time-dependent *z*- and *x*-displacements near the vacuum/crystal and terraced interfacial regions, respectively. As can be seen in the simulations, the initial depth-dependent expansion results in a coherent compressional wave that propagates back and forth along the *c*-axis between the outer layers. At the free edge of the specimen [Fig. 7(a)], stress imparted by propagation of the longitudinal wave results in rapid coupling to in-plane displacements that emanate from the vacuum/crystal interface (i.e., the left edge of the flake in the simulation) owing to abrupt spatial variation in elastic (acoustic) properties. After 100 ps, a coherent wave train of in-plane compressional displacements can be discerned (*t* = 100 ps for *x*-displacement), the emergence of which becomes unambiguous after 50 ps. Similarly, in-plane displacements arise at the terraced interface when the *c*-axis longitudinal waves on either side of the interface become out of phase owing to variations in total transit time. For this type of discontinuity, the in-plane displacements are observed to subsequently propagate away from the interface in both directions and take on a tilted, non-uniform spatial frequency as time progresses.

In order to further quantify the initiation and launch processes and to compare to experimentally observed contrast dynamics, the *x*-displacement simulation data were converted into spatially averaged line scans of intensity (sampled from varying crystal depths) and plotted as a function of time. Shown in Figures 7(c) and 7(d) are simulated space-time contour plots and corresponding Fourier transforms displaying spatial frequency distributions. As with the experimental data shown in Figures 5(b) and 5(c), the simulated space-time contrast oscillations depict the propagation direction and velocities of the *x*-displacements, with the slopes of the contrast bands dictated by the in-plane compressional speed of sound and, thus, the commensurate *C*_11_ elastic constant. Further, the oscillation frequency of the in-plane waves stems from the specimen resonant frequency dictated by the crystal thickness and the out-of-plane compressional speed of sound (i.e., the commensurate *C*_33_ elastic constant). Indeed, the spatial and interfacial dependence of the experimentally observed phonon frequencies are nearly quantitatively reproduced in the finite element simulations.

While the simulations support the hypothesized formation mechanism and coupling behavior of the experimentally observed GHz acoustic phonons, some disagreement remains. First, the absence of low-frequency features in the simulation likely results from a simplification of the actual specimen geometry and exclusion of bends and ripples that give rise to these features in the experimental data [see, for example, the spatial frequency region below 25 GHz in Figure 5(c)]. Second, the model employs a projection along one in-plane axis of the structure and thus does not provide an indication of the wave-front shape in the two in-plane spatial dimensions. For the UEM experiments, the wavefront shapes and propagation directions can evolve and become more complex depending upon the orientation of the two in-plane axes with relation to discrete and discontinuous morphological features. Additionally, the values of the elastic constants can dictate the behavior of the phenomena in the simulation, and these values are often not known for advanced materials or defect-laden specimens. Indeed, future efforts could focus on determining if quantification of the observed acoustic-phonon dynamics can be used to extract the anisotropic elastic properties of nanostructures. Finally, the continuum model applied here is relevant to bulk materials and geometries; while a majority of the presently observed phenomena are well represented in the simulation, future work will determine if a transition to more complex behavior occurs upon reduction of flake thickness (i.e., within a transition region from atomistic to continuum regimes).^25,58,59^

## SUMMARY AND OUTLOOK

In summary, we have described the direct visualization of in-plane acoustic-phonon dynamics at discrete interfaces in single flakes of TaS_2_ and WSe_2_. With UEM bright-field imaging, we have spatially isolated and quantified the emergence, launch, propagation, and interference of phonon wave trains and determined the effects of discrete interfacial regions (vacuum/crystal and crystal/crystal) on the time-varying behaviors. The acoustic waves—discernible in real space via local elastic strain of the lattice leading to modulations of the Bragg condition and the resulting commensurate diffraction-contrast dynamics—arise from interfacial stress caused by initial excitation and confinement of compressional waves along the *c*-axis stacking direction within the specimen-layer boundaries. The transient diffraction-beam scattering-vector magnitudes and angular orientations, as measured with UEM selected-area diffraction, support both the proposed dynamic image-contrast mechanisms and the physical origins of the acoustic wave trains. Guided by these experimental insights, a time-dependent linear elastic finite element simulation of the mechanical deformations was developed and applied, the results of which further support the proposed depiction of the ultrafast photoinduced elastic strain-wave dynamics. Moving forward, applications of combined UEM imaging and diffraction modalities on select, nanoscale specimen regions are expected to be useful in uncovering the physical origins of other phenomena, including (spatial) energy nucleation, propagation, conversion, and decay in myriad materials and composite systems. In particular, one could envision using these approaches to uncover the spatially and temporally varying anisotropic elastic tensors, especially as affected by discrete (and dilute) structural discontinuities. With the spatiotemporal resolutions accessible with UEM, it is also expected that much insight could be generated regarding atomistic-to-continuum structural dynamics, which will aid in informing the continued development of multiscale modeling approaches.

## V. SUPPLEMENTARY MATERIAL

See supplementary material for the control experiments performed and supplementary figures.
